# Why do patients want medication free treatment for psychosis? An explorative study on reasons for applying to medication free programs

**DOI:** 10.1186/s12888-024-05513-9

**Published:** 2024-02-16

**Authors:** Elisabeth C. Klæbo Reitan, Valentina C. Iversen, Henriette Riley, Anne Høye

**Affiliations:** 1 Division of Mental Health and Substance Abuse, University Hospital of North Norway (UNN), Tromsø, Norway; 2 Department of Clinical Medicine, UiT The Arctic University of Norway, Tromsø, Norway; 3 Department of Mental Health, Norwegian University of Science and Technology (NTNU), Trondheim, Norway; 4 Nidelv District Psychiatric Center (DPS), St Olav Hospital, Trondheim, Norway; 5 Department of Health and Care Sciences, UiT The Arctic University of Norway, Tromsø, Norway

**Keywords:** Mental illness, Medication free treatment, Psychosis

## Abstract

**Background:**

A focus on patient’s and service user’s perspectives regarding long-term antipsychotic treatment led to a declaration from the Norwegian Ministry of Health in 2015 to offer treatment without psychotropic medication in mental health as a voluntary option in all four health care regions. In the northernmost region, a 6-bed inpatient ward unit was established, uniquely designed to give people with severe mental illness the possibility to choose such treatment. Only voluntary admissions were accepted. The aim of the present study was to explore the motivation for applying for medication free treatment among patients with several years of treatment history due to psychosis.

**Method:**

We performed nineteen semi-structured, in-depth interviews with persons having at least one admission at the medication free treatment ward during the period 01.01.17 – 17.10.2021. The interviews were recorded, transcribed and analysed using computer-assisted qualitative data analysis software (NVivo). Systematic Text Condensation was applied, with analysis of data from the first interview. Exploration of connections, similarities and nuances was performed through axial coding with continuous comparison of data and memo writing, followed by focused coding identifying core concepts.

**Results:**

All participants had a diagnosis of severe mental illness and a history of use of antipsychotics throughout many years. The motivation to apply for medication free treatment was complex. Negative experiences with medication were described, but also positive. Many had tried to taper off before, but described this as a lonely and difficult process. Five core concepts were formed and developed from the participants’ narratives: 1) Medication experiences, 2) Developing illness, 3) Treatment in general, 4) Social life and 4) Growing up.

**Conclusion:**

The concept of medication free treatment represents a salutogenetic hope for change, closely linked to self-efficacy and an experience of mastery. Potential conflicts concerning guidelines or evidence on medication does not come forward as important. Support from family and professionals is crucial, in contrast to the feeling of being alone when hoping for change. Motivational factors are closely linked to the concept of recovery, where interaction happens on both an individual and a structural level.

**Supplementary Information:**

The online version contains supplementary material available at 10.1186/s12888-024-05513-9.

## Background

Long-term use of antipsychotic medication has throughout several years been debated (e.g. [1–4]). Side effects are well known, but potential harmfulness of medication beyond side effects has also been problematized (e.g. [5–8]). Perspectives from patients and user organisations have been brought forward, pointing at the right to choose treatment with less medication, e.g. user coalition for medication free services [9]. In Norway, shared decision-making has been suggested to increase self-determination and patient safety [10, 11]. A national strategy for reducing coercion pointed in 2012 towards medication free treatment as a possibility [12]. In 2015, The Norwegian Ministry of Health and Care decided to establish medication free treatment facilities within specialist health care [13, 14]. The declaration was met with considerable debate, highlighting the tentative conflict between users’ need for autonomy on one hand and health care services’ obligation for evidence-based treatment on the other. Official treatment guidelines promote non-medical interventions and autonomy in severe mental illness, but also state that psychopharmacological treatment is important and often necessary [15]. Each health care region was free to decide the detailed content of the treatment facilities. According to Standal et al. [3] there were by 2018 twenty-five units throughout Norway offering medication free treatment, defined by Standal et el. [3] as: “treatment free from medication pressure and coercion and not necessarily free from all psychotropic medication”.

There is a long history behind the 2015 declaration in Norway. Already in 1970’s, Antonovsky presented the salutogenetic approach as an alternative terminology to the medical tradition, focusing less on pathology and more on a sense of coherence [16]. In 1977, Bandura presented the concept of self-efficacy, which describes a person’s confidence in his/her own ability to control different life aspects [17, 18]. Subsequently, the concept of recovery has developed throughout the latest decades (e.g. [19, 20, 11, 21]).

The North Norway Regional Health Authority was the only Norwegian region that decided to establish a new, 6-bed inpatient ward unit, uniquely designed to give people with severe mental illness the possibility to choose treatment without using psychotropic drugs. Only voluntary admissions are accepted [22]. In the present study, we aim at exploring patient experiences, expectations and reasons for wanting to apply for treatment at this ward.

## Methods

### Participants and recruitment

From January 2017, the University Hospital of North Norway offered medication free treatment for psychoses and other severe mental disorders in the northernmost health care region in Norway, covering three counties with a total population of 480.000 inhabitants. Eighty-four persons aged 19–63 years (62% women and 38% men) had at least one admission to this ward 01.01.17 – 17.10.2021. Seventy-nine were invited to participate in the study. Invitations were sent out by administrative personnel at the treatment ward. Information was also shared on a notice board at the ward and on the ward’s website. A reminder was distributed in December 2021. Informed consent, clarifying that data will be used in research articles (open access) in line with approval from Regional Committee for Medical and Health Research Ethics (REK) South East Norway (REK 2016/1708), was obtained from all participants. The consent form also state that data will be de-identified (Consent form is Additional file 4). It was verbally specified that statements could be recognized by the individual and those close to him/her but not by others. All participants received a symbolic compensation.

### Interviews

Questions in the interview guide were discussed with Competence Centre for Lived Experience and Service Development (KBT). After piloting the interview-guide during spring 2021, adjustments were made (Interview guide is supplemented).

The interviews were performed in an office or meeting room in proximity to where the participant lived, or digitally. The individual interviews varied in time from 1:50 to 4:15 hours. Sixteen interviews were performed in one session, three were split in two. Interviews were recorded, transcribed and transferred to the computer-assisted qualitative data analysis software NVivo 1.6.1 after de-identification.

### Supplementary information

Supplementary information about former hospital admissions, previously being subject to coercion, diagnosis, medication and treatment plans were collected from hospital patient files. Information on motivation for treatment also includes the patients’ own reports and written applications stored.

### Procedures for data analysis

All the interviews were audio-taped and transcribed manually. The interviews were analysed using Malterud Systematic Text Condensation (STC); a strategy developed and shared by most traditions within qualitative data analysis. The method implies an analytic reduction with specific shifts between decontextualization and reconstruction of the data [23].

The STC-analysis follows four steps: (1) reading all the material to obtain an overall impression and bracketing previous preconceptions; (2) identifying units of meaning representing different aspects of participants’ experience specifically related to the motivation to apply for medication free treatment and coding for these units; (3) condensing and summarising the contents of each of the coded groups; and (4) generalising descriptions and themes concerning experiences with the motivation to apply for medication free treatment [23].

The process of analysis started from the first interview. The inclusion of participants ended when saturation was achieved; the optimal understanding of the participants’ perspective was reached and further data collection did not add substantial new information. In the open coding process, focus was on the specific content in the interviews: What are the participants talking about? Content is both concrete (therapeutic approach and structure) and abstract (experience and assessment). Theoretical memos on ideas and associations were concurrently written down.

Exploration of connections, similarities and nuances was performed through axial coding. Five core concepts were identified, and data condensation around these was further explored using selective coding.

## Results

### Participants

Supplementary to Table 1 on medical use and tapering off: Some of the participants had tried to stop or reduce psychotropic medication by themselves or in collaboration with a physician, without fully succeeding. One had managed to taper down on his own. For the majority a controlled reduction was an important part of the treatment plan after admission to the ward. Structured tapering plans often range from months to years. For the majority there was a reduction of antipsychotics during treatment, but the picture was heterogenous. Four participants increased their dosages of antipsychotics due to worsening of mental symptoms. Dosage and type of medication was developed in collaboration with the patient’s general practitioner, psychiatrist and/or local health care workers as far as possible, but the patient was considered to be the main person of expertise.

**Table 1:** Characteristics of participants (*N* = 19)

Gender	14 women, 5 men
Age	23 – 53 years (median = 38)
Years with illness	8 – 28 years (median = 15)
Number of admissions	1 – 30 admissions (median = 8)
Former experience with coercion	11
Work/income	14 received disability benefits (50–100%.) 2 had been receiving disability benefits earlier 4 worked or studied 6 were active in voluntary work 1 unknown work/income status
Social and familial	8 had children of their own 7 were cohabiting 12 lived alone (3 with shared care) 7 reported having animals
^b^ICD10 diagnosis at the time of the interview^a^	11 had F20–29 (Schizophrenia, schizotypal and delusional disorders) 5 had F30-F39 (Mood affective disorders) 4 had F40-F48 (Neurotic, stress-related and somatoform disorders, primarily PTSD and social phobia) 1 had F60-69 (Disorders of adult personality and behaviour) 1 had F10 (Alcohol related disorders) 1 had no diagnosis
Substance use	4 reported having had drug addiction at some point
Medication	All had been using antipsychotics for years 13 had previously had regular used antidepressants and/or mood stabilizing medication 15 had used sleep medications and/or sedatives benzodiazepines 12 had somatic illnesses for which they continued using medication

^a^Several had more than one diagnosis

^b^During the course of treatment, 14 participants had no diagnostic adjustment whereas 5 had their diagnosis adjusted or removed

### Wish for medication free treatment

**Fig. 1:**
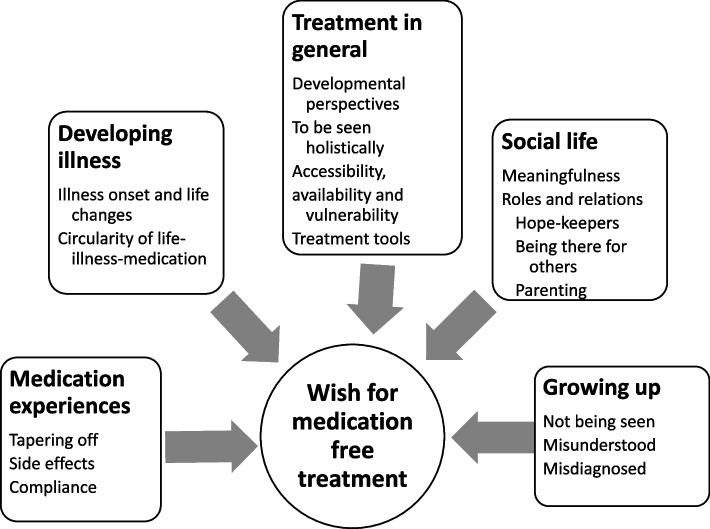
Concepts associated with the wish for medication free treatment

Five core concepts were formed based on participants’ narratives (Fig. 1). The concepts are influenced by one another, nevertheless they appear as autonomous entities in the analysis. Numbers in brackets behind citations refers to the participants (1–19).

### Medication experiences

The content in this concept were 1) Tapering off 2) Side effects 3) Compliance.

For many of the participants, the motivation for tapering off psychotropics is connected to an experienced lack of effect, often over a long period of time. In turn, this links to the complexity of understanding symptoms, e.g. the fear of being misdiagnosed or not getting the correct treatment.

*I’ve never seen any effect but lot of side effects … Have not been met on the lack of effect. It’s been difficult (2)*

*Medication for medication: A new medication to calm down side effects from another. I remember being tired of that medication … It was a vicious spiral (10)*

*Then we tried it for two months, it did not work. Then we tried it for two months. And it did not work. Then I got ECT. And it did not work. Then I got a new diagnosis. Then everything changed for the better … Nothing worked, and I didn’t understand it myself, eh, because I didn’t understand that what I saw and heard was not normal … It took some time before they understood that too because I didn’t understand that they were wrong … That is why it took so long to get right diagnosis (4)*

Doubt and ambivalence are emotions related to tapering off. Some are afraid that normal functioning such as being a parent or going to work, might be disturbed if they taper off and, for them, these aspects are valued higher than not using medication.

*I feel safe I will not get psychosis and things like that, as long as I take my medication … I have medication… (olanzapine) in the bedside cabinet for when I’m going to sleep, long before work … There is no reason to change what works … I’ve felt getting tired from the medications, negative things. Now I see that I’ve managed getting through, things work fine and the medication helps me not getting delusions and psychosis and being in a state of mind where it’s impossible to concentrate about anything else (5)*

*For me it’s more important having a job and my child than weighing 10-20 kg less (9)*

Most participants have experiences of tapering off, and they all express the need to do this in a safe environment and in collaboration with health care personnel. Many feel they have been alone in wanting to reduce medication. For them, the new possibility that came with the treatment ward fostered an opportunity to try tapering off within safe conditions, and not being alone doing so.

*I had reconciled myself with that (sleeping much) … it was sad. Then I heard about the ward … Started a process that perhaps there was a life outside of the couch (4)*

*Before I was told I would have this diagnosis, would have to receive help and use medications for the rest of my life. Limitations all the way …. Every time I got what were side effects, it was seen as worsening of illness. And the dosage increased (2)*

Those who have tried without support express the process as difficult, for some even scary.

*The way I stopped taking medication (after 17 years) is not to recommend. It was a silent protest … I quitted immediately … I became very ill (13)*

*I thought that if I just got to go to school, start doing something, exercise, and things like that, then everything would be ok… This kind of traumatic reactions start to kick in when you are off medication. It all became too much… I thought it was an earthquake… And I thought that it was the medications that stopped my memory… that medication was just a lid (11)*

Some have experienced more trouble from side effects than from the illness.

*Personally, I feel antipsychotic medications are OK used as fire- extinguisher, but not as permanent medicine. I have had more trouble from side-effects of antipsychotics than from the diagnosis itself. Medications only alleviate symptoms, not what is the real problem and what are daily problems. I wish I would learn to live with the challenges I have without having to resort to medications. I hope I’ll find strategies of mastery (7)*

*I expected, I think, when medication was tapered off, then I could, everything would be fine, I could start working, living and live a completely normal life (18)*

One of the most frequently mentioned side effects is gaining weight.

*I have gained so much weight and have so much fluid accumulations and felt that I’m in a straitjacket (16)*

*Using … (olanzapine) I gained 50 kg. Then I got cholesterol and water in my body (12)*

*I gained 30 kg in one year … apathetic or having no feelings … indifferent … tired and difficult to move because of muscle stiffness … I was still depressed, just with side effects too (8)*

Increased tiredness and need for sleep are factors frequently described.

*Side effects were never an issue … every time I got side effects, it was interpreted as worsening of illness, increasing dosage … sleep, I slept a lot … it was understood as beginning of psychosis because I slept and was tired … The higher dosage, the more tired I became (3)*

*It means lying down for 18 hours a day … having alarm clock half an hour before husband and children arrive from work and school, to make it look like I’ve been awake all day. Which I have not been. I’ve been lying doing nothing, sleeping, being sedated by medications. Not having anything to tell at the dinner table. ‘What have you been doing, mum?’. ‘Nothing.’ I haven’t been eating lunch, nothing (4)*

Emotional side effects are described by many, including fear of irreversibility and of losing contact with creativity.

*Feeling tired, feeling being distant … heaviness and laxity …. Thoughts being absent ... I simply have to learn feelings and learn reactions. I do not have them, I have to learn them. I have to learn how to react (10)*

*The patient experience side effects from medication in form of bodily twitching, is afraid of getting diabetes and that medications might give brain damage. The patient feels trapped in medication, stigmatized and being outside of autonomy. Want to master disorder without medication (*medical record*, 1)*

*I have had many strange side effects, like; I like drawing and side-effects have made it impossible for me to be creative … which destroy my quality of life (15)*

Distrust and loneliness are emotions expressed as related both to side effects and to miscommunication.

*When meeting health service having side effects like spasms and tics, no one listened to my family or me … They meant I suffered from epilepsy (10)*

In the personal application (medical record) the same participant writes: *When the therapist turns into voices because nobody of the closest can relate to what I’m going through… Being met with distrust, misunderstandings and secrecy makes me alone and lonely (10)*

Motoric side-effects mainly relate to a lack of control over body movements.

*I had problems keeping the lips together, had to change head-pillow every night because of drooling … I noticed that I lost control over my body, it was strange … (later in interview) I had to think of put the right foot in front of the left to be able to walk (18)*

*Suddenly it was jerking in one arm. Suddenly it was in the face. The shoulders and the head. There were these tingling in the legs, I guess they call it restless legs (10)*

“Other side effects” frequently involves the interaction between side effects and symptoms of illness. One example of this is when some describe the negative developmental consequences of using medication in adolescence.

*It’s helped in periods but I’ve been overmedicated… I was not strong enough to say no … I got worse of medication… I was out of society, out from everything because I was so tired (6,* had used medication for 15 years*)*

*It started quite early… I’ve been thinking that (pause)… somehow I didn’t become a grown up before I started struggling … The brain is perhaps not fully developed in women before the age of 24/25, so I got lost somehow, I was much detached … I felt that health service didn’t take care of me … The onset of psychosis came when I finished (having had eating disorder and mononucleosis). Or, not having psychosis, but getting drops of psychosis (11)*

Social and societal attitudes are related to compliance, but also to stigma. Further, being accepted as ill means taking medications.

*It’s stupid having all these needle marks on my arms because I have to take blood test every month for years … It’s taken so many years of my life … not having children of my own … much isolation, much stigmatizing in society … and the attitude towards myself of what I deserve and not, how people look at you and not being able to build oneself up (10)*

*You can’t really be ill when you don’t eat medications. I don’t know how many times I’ve heard that … If you are ill, you have to take medications … It seems people think you can’t be ill and be disabled and not be eating medications (13)*

Compliance is connected to their own understanding and interpretation of symptoms. The relation to health care personnel is important, especially to the psychiatrist or therapist.

*If it is introduced as an easy solution, like, when you enter and are psychotic, you want to grab the first thing … And if they tell you that medication is going to get you well, then there it is (11)*

*Things have changed and depends much on who medicate, you know, who gave medications and how they presented it for me… I’ve become older and have perhaps gained experience and think: Take a pill and get well. But it’s not really working like that (laughs) … I guess I’m like everyone else, wanting an easy solution sometimes … Perhaps it’s about hope, that someone is better at selling hope together with the chemical package that comes around, while others are more like it’s not easy to know what lies behind… In the beginning I believed strongly in medications because the psychiatrists I met, believed strongly in that, and I guess, they saw a great change in me being on and off medications (11)*

*Most of the time I listened to the doctor… because it’s most profitable … sensible (1)*

*And you know, he is so good with relationships, we do have a good relation … what is good … we exchange (reflects upon having been ill for years). The psychiatrist has thought me, and teaches me, that I’m a human being (10)*

Some report that medication is a first-hand solution, but also the only available solution.

*I feel that psychiatry doesn’t have anything else to offer than medications … And I do miss that. If you scream and shout and need something. Yes, then you are admitted, and you get medications (13)*

*In the beginning I got lots of medication. No information about side effects. When I got them there were not so much … not so much collaboration. I got a treatment that didn’t fit and that was all you got. Much medication was what I got. That was not much treatment, really (15)*

Several express that medication do help, and some feel more vulnerable and more susceptible to violations without medication. Yet some are disappointed because medication does not cure the underlying problem; *Medication is just a tool* (19).

*Medication has had a calming effect. It’s challenging to adjust without them* (medical record, 10)

*Last year I finished using antipsychotics after having used…(clozapine) for 19 years … then I had (breathes in, heavily) then it all came back. All of it … So… (clozapine) had only lowered them in a way and not been treating (10)*

*Medication…someone need it … but you do not work with the real challenge (8)*

*I think it’s the medications that rules your life, somehow. (laughs) That somehow regulates it … And I’ve accepted that probably I’ll have to use them the rest of my life (laughs). I was thinking much about that medications decide, -and they do! What I’ve understood is that there is a lack of something the brain doesn’t produce enough of, I’ve heard … When I don’t use medications, my brain works so fast … The substance the medication gives, is stopping my train of thoughts. Slows down. Make it work normally, not being chaotic (14)*

*I’ve eaten medication for 15-16-17 years … I realised they had no longer effect. It was too much. Too much side effects and too much losing course and direction. I think it was taking medication to lower side effects. Not to limit the illness. They did help in the beginning, the medication. I’ll say so. I don’t think I would have been here today if not for medication (13)*

Switching medication and questions of dosage is also related to compliance.

*Yes, I had lost myself. I can see, I didn’t want to become like my (relatives), but I did. Almost never being outside. I could get out sometimes, with friends, but didn’t participate. Never went to the cinema, never doing anything that I used to do before entering mental health services. Then after switching from… (perfenazine to quetiapine) I changed! Suddenly I started showing feelings like crying. I began looking pretty upbeat (17)*

*I have noticed using high dosage make me zombie-like, little in contact with myself. Being down. Loosing yourself and that is very creepy (later the participant says). I tried several times reducing gradually, very carefully. It was fine until I came to the last small little dosage. I thought it would be fine since it was fine on the smallest dosage that should not really have any effect … But that minimal dosage that should not have effect on me – it did have an effect … I became ill when I quitted. I became psychotic. So, I’ll realise that at least I need a small dosage … It’s a little bit sad (15)*

### Developing illness

Developing illness can be related to stressful or traumatic life changes. Others describe onset of illness as unforeseen.

*I was on maternity leave, a different life from what I was used too, became depressed … behaved different from usual (7)*

*I got ill at the age of 30, had never been ill before … I became manic, with psychosis, when first getting ill (9)*

*I didn’t understand that what I saw and heard was not normal … took time until they understood that too. It took a long time until I got right diagnosis (4)*

*I didn’t accept being ill and I was thinking ‘now I’m going to get better’. It was like this: I was really not ill. I was just not good enough being healthy. I had to work harder (15)*

Several of the participants explain how the illness has developed in relation to medication, and describe areas where they think medication has actually worsened their symptoms.

*I became more tired and experienced burnout … dosage of medication increased … sleep disturbances got worse (1)*

*This hospitalisation was the beginning of it, but I guess I’ve been bipolar, really, more or less … having periods with illness and being on sick leave from work … I think I have been depressed. But I do think this forced hospitalisation triggered the bipolarity (13)*

Several participants use the term “being in storage” when describing treatment, and some are explicit on a circularity between illness and medication compliance.

*Been in hospital, taken medication and feeling good about myself, thinking: ‘nothing is wrong with me so why eating medicines?’ and stopped taking medicines, gradually becoming ill again… I need medication not to have troubles (5)*

*The help I’ve received before has been like firefighting … when things have been really rough, I’ve had to be here (at hospital). I feel it is firefighting. I’ve felt like being a patient in a revolving door. Coming back again and again … At a storage location … were they were nice and sweet. That was not the problem. But there were no goals and no meaning in it what they were doing … just being there … and getting lots of medicines (4)*

### Treatment in general

When participants talk about treatment experiences, we especially searched for expectations they think will be different with medication free treatment.

Some report meeting too little focus on developmental perspectives.

*I have no doubt that people meant well for me, but it has been a kind of top-down attitude especially from psychiatrists. I’ve felt that my right to determine something for myself, has been restricted to time (when to) don’t give attention to this and that. Almost like training a dog (laughs) (2)*

There are reports on lack of dual attention toward mental and physical health, individually or at a system level.

*People have just asked me what has been going on in my head, never my body (4)*

*I guess that your physical health is what you bring for the GP (11)*

There may be too much focus on specific symptoms.

*There was only focus on the symptoms I had … and what to do to get rid of the symptoms. There was no talking about how are you doing, what’s going on in your life... never a focus on all of me (3)*

*Feels like being at a storage place … stabilisation … and focus on medication at that time. I have schizoaffective disorder, but what really bothers me is depression (8)*

Kindness is mentioned in a thankful manner together with appreciation of meeting a system that knows something about one’s history.

*I feel that all help I’ve got from health services has been very good. And I’m very grateful for all the support and help I’ve got and (swallows) and, yes, both health services and NAV. I’m … Incredibly grateful for all, all facilitation and guidance, guidance and, and help and, and support. It’s been very important to me (5)*

*I sat smoking instead of being allowed going out, because they didn’t know me … But at my last admission there it helped more because then I had a medical record. Before I had not, then I had only a medical record for my physical health (12)*

One aspect of accessibility and availability is having to fight to find someone who can help with what you want help for, and accordingly the qualifications of the therapist. Having the same therapist over time is mentioned, like one participant who express: *Both having change of environment and having a therapist over time has been good (17).*

There are also several reports on lack of continuity and help getting guided forward.

*I have been admitted to hospital before, for emergency treatment … When you are discharged, it is to nothing … Then, you are at home, you know … And then there are no follow-up, no further prevention (9)*

*The therapist couldn’t help me with the rest of it … I had to make effort to get in touch with a psychiatrist, saying I wanted to change therapist (7)*

*It was the taxi-driver who guided me to the emergency unit. And that was not really his job … If you are alone and have no surplus, how can you then ask for help? … It was my cohabitant who called to get me admitted. I didn’t manage to speak … This was even though I was already in the system having had psychosis before. How difficult can it be getting in there for the first time (15)*

One participant had experienced both being a patient and a relative and expanded on accessibility of services based on being understood or misunderstood.

*It is very difficult getting help when the one at the emergency unit says you are not psychotic. They said: ‘He’s not psychotic, that is for sure.’ And then – it was exactly what he was … They say: Don’t be afraid of seeking help, but then, when you don’t get it, why ask for it, really … It’s been much despair … The emergency unit sent me to GP and GP didn’t want to do anything… it was very difficult (15)*

The interpretation of availability varies. Most have extended experience from different forms of therapy. One participant (9) emphasises in the personal application*: being with other and by oneself, mindfulness, relaxation, music and different therapeutic interventions.* Another participant (7) also includes *mastery, psychoeducation and mindfulness*. A third (6) emphasises* cognitive therapy, physical activity, setting personal boundaries, psychoeducation, therapy with animals, trauma-therapy, making art and dieting*. Being in a vulnerable position regarding what is available, is perhaps most clearly expressed in a quote on receiving medication free treatment.

*I’m worried that I will not reach the finish line before the state says this will be too expensive. Or too irresponsible (2)*

Some describe that life might take a surprising turn, such as getting a dog or experiencing someone taking a chance in believing in one. Some validate joy and distractions and some mainly want something different from interventions they feel have silenced them.

*I have felt that my mental health has not been taken care of in the municipality. I had a psychologist working private, I have been rejected twice from local outpatient clinic… For months I was just lying there in bed* (getting ECT, experienced it like traumatic coercion with memory loss. self- harming and anesthesia that didn’t work)... (later in interview) S*tabilisation…yes, getting out of bed, joining activities … Getting one conversation a week. Perhaps two… Then I got a dog! It has come to my attention afterwards; There were a mental nurse that said that if I was not capable of taking care of the dog, she would do it … I do have met angels like that in the system. So, they are there (laughs) (6)*

*I have been trying to do things that I like, things that bring joy … In addition to that I try getting outdoors every day. Sometimes it helps, many times it doesn’t help. (*medical record*, 8). For me it was to distract myself (interview, 8)*

*I became indifferent, it was like the psychologist and these knows-it-all, made me be silent and not, yes, complain … Being admitted to the local bed-unit, the first thing you were told, was not to talk about what you suffered from and what medication you were using … There were two tables for eating, and you had to sit were your room number was written. If you sat down at the wrong table they yelled at you. And you were not allowed talking by the table. There were to many strange rules (about social training at a café) Being there, at the café, I got very sad. Suddenly. I don’t know what happened, I just started crying. I couldn’t stop. Then the employee told me to toughen up because she had been looking forward to this all day- eating out. I didn’t manage toughen up and we had to go back. This has made me not being able to cry again (even at funerals) (18)*

### Social life

Life aims differed widely. For some, having a regular job is very important, whereas for

others their main ambition is the management of daily life activities. Importance of relationships is frequently mentioned. To have someone close (family, friends, lovers) who can carry hope or have common goals, is expressed as crucial in the process of recovery, change and motivation. One participant named this special person *My obelisk … my mountain … a companion! Solid. All the way (10).* Others expressed both trust and mistrust in helpers.

*I thought: Doctors have lied to me … I understand! I don’t have to be ill the rest of my life and not be able write music (1)*

*I have bad social network. I have a lot of friends and acquaintances, both on mail and chat and to do things with … But, close ones, I do not have. I’m working on it (13)*

*I am very fond of my friends, I like my family very much … For a period of time I was worn out, forgotten, lying on the sofa. … I’m very thankful for the ones not giving up on me (laughter), who didn’t stop calling, who didn’t stop … I feel I have something to catch upon (4)*

*I never liked medicines. None in my family has approved either. They have been a great motivation for me to stop using medication.* (Later:) *My goal is getting back to work (16)*

Some describe a muted access to meaningfulness when being on medication.

*There are times I don’t do much. I like drawing, painting … I have been doing it on medication too, but it has come more pleasure into it now … When I’ve been using medication I have almost not … felt any liking for doing anything … have not felt any joy in anything. Like watching a movie: I could watch only half of it (and then) I had to stop … (16)*

For many, a need to be able to use one’s past in being something for society or other persons is a strong motivation for wanting change. Some engage themselves, some want to do so but cannot yet and some explicitly protect themselves from social arenas and commitments.

*I want to be the one who care… the one who starts writing list for Christmas in August just to make sure that everyone gets what they do want (4)*

*I try being a voice for others. I feel that when I have been open about my illness, like in the newspaper, it’s not that I want to fold out my history … but I think it’s important for many more… (tells about feedback:) I said that I missed being important for someone. Then he started crying and said: You have been very important for me (9)*

*I have always had a wish of getting back into the system, and contribute with something else than being a patient. Being allowed sitting on the other side of the table (10)*

A particular awareness of relations and roles regarding parenthood is expressed.

*I’ve been talking to (my child) … And I’ve asked if she is sad that mom was not present (during childhood). (She said) … I had father and granny, so it’s … Just like that! She thought it was ok (4)*

*I have great worries that… I’ll never get totally … well enough to have a child because I get tired from everything … It’s quite a heavy burden thinking of … there are lot of things I want to do, and then (pause) I can’t (6)*

*What I’m afraid of is losing my child … having to worry about what I tell health personnel (7)*

### Growing up

Childhood experiences related to not being seen, understood or getting help on time are described. Several have experiences (like neglect, bullying or been sexually abused) that has implicated a lack of security, which in turn has prevented them from seeking help, to trust that help is possible or that the help offered is what they actually need.

*I think what I would have needed was that someone had (pause) understood that I (pause) perhaps didn’t act like a normal child … And that someone took hold of it, a grownup taking hold … saying that this child needs more support (later) I have heard that someone I went to school with, thought that I had special need or that I was mentally disabled … because I was so (pause) strange (6)*

*The therapist said she thought that I had been having problems all my life … because I was not very old when the assault happened (12)*

There are stories of being misdiagnosed or misunderstood as psychotic during childhood or adolescence.

*I had a stressful childhood (tried to commit suicide 8 years old). It might be difficult to differ on what is one and what is the other (2)*

*I had always been a worried and sad child, perhaps not always, but it developed like that. The reason why I was … referred to treatment by a psychologist … was because mum and dad needed help on how to help me because I was so sad and I had separation anxiety, was afraid of fire and had very low self-esteem … because of bullying among friends … My bad thoughts about myself, I expressed as voices … hearing … I have never had any psychosis, have never been admitted to any other ward (3)*

*I have been receiving wrong treatment, throughout the years, getting wrong diagnosis … The first was Asperger … and then there has been different personality disorders. (not schizophrenia?) That too (8)*

*Since I always have been seeing dead people and been able to talk to them since I was born, I don’t know anything else … he (psychiatrist) meant I didn’t have ME and that I was psychotic and had been, obviously, for a long time. (laughs). After being there for a while, four days, I got the diagnosis of paranoid schizophrenia and got treated as such. … I have never felt psychotic (18)*

## Discussion

### Motivation for change

All the five core concepts are somehow connected to a hope for change. The perspective that something different might be done has a huge influence on applying for admission to this treatment. Some see an opportunity to counter stigma about mental illness, both self-perceived stigma and stigma from the surroundings, and describe the ambivalence towards illness, treatment experiences and wellbeing as a vicious spiral. For some participants, their own experiences dominate, whereas others underline the importance of an alternative professionalism behind the establishment of the medication free treatment that they can lean on. They also explain that this is somehow supporting a longing for coherence. This relates to Antonovsky’s concept salutogenesis [16], which focuses on what make people survive and grow despite the circumstances, taking into consideration not only what is possible to do, but also whether it is meaningful to do something about it – if is it worth the effort. Central in coping well is a high degree of sense of coherence, operationalized as comprehensibility, manageability and meaningfulness. From Bandura [17, 18] we borrow the concept of self-efficacy, resting mainly on four sources: Mastery experiences, observational learning (vicarious experiences) and verbal persuasion, as well as physiological and affective states. Bandura explores [18] the complexity of interaction between these and a non-linearity in weighting them.

Both sense of coherence and self-efficacy might also be understood as part of the recovery process as defined by Dell et al. [21]; A transformation from a negative identity state to a state of positive psychological well-being. In our study the sense of coherence is fragile, but present, much like The Invisible Child in Tove Janson’s book [24]: Being present but hidden until one is loved and connected to someone else.

To some degree, our findings are in line with other studies. In 2018, Standal et al. concluded with three meta-themes on why patients want medication free treatment; negative side of psychotropics, struggle getting treatment without medication and that compliance to medication can be in conflict with values, attitudes and beliefs [25]. The relational aspects we find are also in line with Ødegaard et al. [26], who underlines that an acceptance of personal coping strategies and personal responsibility for the recovery process might give room for a higher acceptance level for discontinuation of medication. Yet to a greater extent our data point at a lack of continuity, and a narrow space for addressing side effects.

### Comprehensibility

Comprehensibility can be understood as a way of understanding that builds up from experiences; that there is correspondence between what happens and what is done. Comprehensibility is expressed in all the core concepts. Many of the participants present challenging experiences from childhood and upbringing. Some have experiences of not being understood, or even misunderstood or neglected, at an early age, and not being met with empathy. The narrative of their life with mental illness can be seen as reinforced by the vulnerability created by these early life experiences. Comprehensibility is hence a crucial factor at the meeting point between patient and health service providers.

### Hope and manageability

In line with Antonovsky’s third concept on motivation, the presence of hope might be interpreted both as comprehensibility and manageability, both aspect important to consider before concluding whether it’s “worth a try”. Hope is related to both self-efficacy, building on one’s own experiences, observational learning and verbal persuasion (when others verbally express their way of understanding). Some express having had hopes of being cured by medicine and hence being disappointed when symptoms have reappeared after tapering off. Some state that medication does not do anything with “the real problem”.

The participants express the feeling of aloneness throughout several years. Some express that even talking about this subject with professionals was difficult and that the easiest solution was to go along with standard procedure; taking medications. Attempts to taper down is described as very hard, and the need for having safety and backing during this process is an important motivational reason for applying. Many reports a stressful lack of control, hence the vicious cycle consisting of symptoms – medication – ending medication – getting ill – medication again. For many, the side effects and the subjective experience of lack of intended effect of medications are important motivational factors.

All participant explicitly express that it is meaningful to try, independent of whether it has been successful or not earlier in life. Getting the opportunity, that someone believes in the possibility, is of importance.

### Relations to others

In 2020, Blindheim addressed challenges relating to complexity concerning coercion, medication and relations/roles between patients and professionals [27]. This is also reflected in the present study. Relational experiences linked to medical treatment, even coercion, are by some described as necessary, while others find it mainly problematic.

Many express the burden of being alone with the thought of living a life without psychotropics. Some have, however, been “allowed” to try, in cooperation with a psychiatrist or a GP. Some have extended experiences of trying several new medicines, and some has not yet found what works well enough when taking the side-effects into account. Everyone talks about the relation to helpers, availability of services (both in general and for long enough time) and accessibility to knowledge and resources. Family relations also have a major impact on being seen; simply to be cared for and to carry a hope for change.

### Experience of mastery

For the majority, their experience of personal mastery is a crucial factor for finding motivation to apply. This might seem obvious, but is worth resting upon. Even if ambivalence and need for secure frames and social support is clear, the effectiveness of medication is, somewhat surprisingly, muted in the narratives. Some are explicit on the positive effect of psychotropics, but for most there is a hope (from subtle to clearly stated) of managing life “good enough” without medication. This may point towards a deeper understanding of what ‘’medication free treatment’’ represents; a belief in mastery and change. For many, to experience one’s mastery is to find a balance between symptoms and what to do about them – both medically, behaviourally and mentally. Mastery is also to be able to do what they express as giving, such as creative activity or being a good parent. At the same time the sorrow and lack of mastery is present when engagement or participation is hindered.

## Strengths and limitations

Medication free treatment is heavily debated politically and academically, which may have influenced the participants' narratives and also the researchers’ interpretation of these. Further, participation required extensive interviews, which could have hindered patients with e.g. more symptoms or patients with other motivations for applying from participating. This may have affected the representativeness of the narratives’ content. However, a strength of the study is the inclusion of a total of 19 participants, ensuring a broad supply of data that allows for extensive variety in the narratives.

## Conclusion

The motivation to apply for medication free treatment is complex. Negative experiences with medication are described by several of the participants, but also positive. The concept of medication free treatment comes forward as a salutogenetic representation of a new hope for change, closely linked to self-efficacy and experienced mastery. Potential conflicts concerning guidelines or evidence on medication are not brought forward as important. However, having someone as support, both family and professionals, in contrast to the feeling of being alone in daring to hope for change for the better, is of major impact when applying. The motivational factors described are close to the concept of recovery, where interaction happens on both an individual and a structural level. A person’s possibility to choose depends on opportunity resting on good relations and being allowed to be in a process based on one’s own experiences.

### Supplementary Information

**Additional file 1: ** Interview guide – translated to English for the purpose of this paper.

**Additional file 2: **Side effects. The main content of the concept were weight, sleep, feelings, motoric and other (more rare symptoms mentioned by one or a few).

**Additional file 3: ** Treatment in general. The main contents in this concept are developmental perspectives, to be seen holistically, acceccibility and availability and tools. In the figure you find them explained.

**Additional file 4: **Consent form (translated from Norwegian to English for this publication). Medication free treatment in Northern Norway – how is it experienced by users and practitioners?

**Additional file 5: **About the reference group.

**Additional file 6: ** Additional information about *the population from which our sample is derived*, that is; patients at the ward from January 2017 – October 2021. Information received from the Medication free ward quality register approved by Data Protection Official at University Hospital of North Norway (UNN) Number 02075.

## Data Availability

The datasets used and/or analysed during the current study consists of in-depth qualitative patient interviews not publicly available for confidentiality reasons. Information about the dataset and analysis are available from the corresponding author on reasonable request. The interview guide is available as a supplementary file.
