# Supplementary material for: Why do patients want medication free treatment for psychosis? An explorative study on reasons for applying to medication free programs
Source: BMC Psychiatry. 2024 Feb 16;24:127. doi: 10.1186/s12888-024-05513-9 (PMC10870549; doi:10.1186/s12888-024-05513-9)
Supplement: Supplementary file 1 — Additional file 1: Interview guide – translated to English for the purpose of this paper. [file 12888_2024_5513_MOESM1_ESM.docx]

**Interview guide**

**– translated to English for the purpose of this paper**

**Instructions for interviewer:** Notice and make notes. If you don’t get concrete answer, then elaborate e.g. how was this yesterday. Be aware of if words you use are understood.

**Introduction:** Thank you for wanting to participate in this interview regarding a research project on drug free treatment offered and how it’s experienced. We will probably need 2x 45 minute and can. We can talk continuously or split it up in breaks if you do prefer that. All interviews are recorded and will be fully transcribed. This is *not* part of the medication free wards work, and what you tell, is not going to have any influence on your treatment – regardless of whether it is complaints or compliments. When information from the interviews will be used in analysis, they are de-identified to make sure it’s not easy to know that you have given your answers. (says something about some self-report forms) I’ll start asking about experiences from treatment, need of support, history of illnesses and something about who you are. Let’s start talking about the treatment at the medication free ward, tell about this. If you please, compare this to experiences you have from treatment before.

| Content of treatment |
| --- |
| Does it work – belief in it/how you notice that |
| Help for what you want to be helped with (the right help, support) |
| What are your experiences with psychopharmacological treatment  (has it changed/ is it related to effects, side effects, dosage or other) |
| Did treatment contribute to support or strengthen you network |
| Cooperation between services (do they receive service from different service institutions, is it expressed need of collaboration between them, does it occur) |
| Themes  Were they part of treatment, how, are they influenced by treatment   - Autonomy - Vulnerability - Mastery - Change - Alliance - Motivation - Recovery - Other (ask) |
| Continuity and quality |
| Availability (how did you get in contact with the ward) |
| Expectations about what the treatment might lead to regarding:   - Network - Autonomy - Vulnerability - Mastery - Change, ability to - Mental health (what is this for you) - Physical health (what is this for you) |
| Evaluation of the treatment: How did you experience it (something very good, something very bad) |
| Background information   - Age - Activity/work - Social status - Network (meaning, whether getting support, role) - Interests - Has anyone expressed something about you wanting this treatment - Illness history (for how long have you struggled with what you seek help for at the ward) - Number of admissions - Medications - Any thing else you want to add |
| About health in general   - Do you worry about health issues (how do you cope) - How is your health (your evaluation) - What do you think about your sufferings – how do you understand them |
| What is important in your life   - Life-quality (what is, which part of life) - Being satisfied - Did you expect them to change due to treatment |
| Anything else you want to add |
| After ending interview: Do you want to say something about the interview |
