# Supplementary material for: Why do patients want medication free treatment for psychosis? An explorative study on reasons for applying to medication free programs
Source: BMC Psychiatry. 2024 Feb 16;24:127. doi: 10.1186/s12888-024-05513-9 (PMC10870549; doi:10.1186/s12888-024-05513-9)
Supplement: Supplementary file 2 — Additional file 2: Side effects. The main content of the concept were weight, sleep, feelings, motoric and other (more rare symptoms mentioned by one or a few). [file 12888_2024_5513_MOESM2_ESM.docx]

Side effects. The main contents of the concept were weight, sleep, feelings, motoric and other (more rare symptoms mentioned by one or a few).
