# Supplementary material for: Why do patients want medication free treatment for psychosis? An explorative study on reasons for applying to medication free programs
Source: BMC Psychiatry. 2024 Feb 16;24:127. doi: 10.1186/s12888-024-05513-9 (PMC10870549; doi:10.1186/s12888-024-05513-9)
Supplement: Supplementary file 3 — Additional file 3: Treatment in general. The main contents in this concept are developmental perspectives, to be seen holistically, acceccibility and availability and tools. In the figure you find them explained. [file 12888_2024_5513_MOESM3_ESM.docx]

Treatment in general. The main contents in this concept are developmental perspectives, to be seen holistically, accessibility and availability and tools. In the figure you find them explained.
