# Supplementary material for: Why do patients want medication free treatment for psychosis? An explorative study on reasons for applying to medication free programs
Source: BMC Psychiatry. 2024 Feb 16;24:127. doi: 10.1186/s12888-024-05513-9 (PMC10870549; doi:10.1186/s12888-024-05513-9)
Supplement: Supplementary file 4 — Additional file 4: Consent form (translated from Norwegian to English for this publication). Medication free treatment in Northern Norway – how is it experienced by users and practitioners? [file 12888_2024_5513_MOESM4_ESM.docx]

REQUEST FOR PARTICIPATION IN RESEARCH PROJECT

# MEDICATION FREE TREATMENT

This is a request for you if you want to participate in a research study where we want to examine the newly established treatment offer for people with severe mental illness in The Northern Norway Regional Health Authority. We ask you because you have experiences with this treatment offer.

WHAT DOES THE STUDY INVOLVE?

The study includes participation in an in-depth interview with a researcher. Topics discussed are mainly your expectations of and experiences with the treatment offered. The interview will last for 1-2 hours. You will also be asked to complete a structured questionnaire.

We also ask for permission to gather information concerning diagnosis, health evaluation and former treatment history from medical records and from the in-hospital quality registry for medication free treatment at UNN HF. We further ask for permission to gather information from your general practitioner concerning number of consultations and prescribed medicines during a two-year period before admission to the medication free treatment ward. In addition to individual interviews, the study will include interview in groups with practitioners/employees.

The study takes place at The University Hospital of North Norway, with financial support from The North Norway Regional Health Authority.

POSSIBLE ADVANTAGES AND DISADVANTAGES

Possible advantages from participating in the study are that the results might lead to a better and more comprehensive knowledge of expectations to and experiences with a medication free treatment offer, which has not been established elsewhere. Results from the study may lead to the generation of new hypotheses for further research, and will be important for development of healthcare services for the group of patients you represent. You will not have any disadvantages from participating in the study, except that it may be somewhat time consuming.

VOLUNTARY PARTICIPATION AND POSSIBILITY TO WITHDRAW CONSENT

Participation in the study is voluntary. If you wish to participate, sign the declaration of consent on the final page. You can withdraw your consent to participate at any time and without stating any reason. This will not have any consequences for your treatment.

If you withdraw from the study, you can demand to have the collected data deleted, unless data has already entered into analyses or have been used in scientific publications. If you later want to withdraw or have any questions regarding the project, you can contact the department or project manager Elisabeth Reitan.

WHAT WILL HAPPEN TO THE INFORMATION ABOUT YOU?

Information gathered about you will be used in accordance with the purpose of the study as described above. You have the right to access the information that has been gathered about you and to correct any errors.

All information will be treated without name and date of birth or any directly recognizable information. A code number links you to your data through a list of names. Only authorised personnel connected to the study will have access to this list of names and can trace the information back to you.

The project manager is responsible for day-to-day operations of the study and for ensuring that information about you is handled safely. UNN HF (the hospital) is responsible for data processing. Information about you will be anonymised or deleted no later than 2043.

It will not be possible to identify you in the results of the study when these are published.

APPROVAL

The project is approved by the Regional Committees for Medical and Health Research Ethics South-East (REK sør-øst) (2016/1708).

## CONSENT FOR PARTICIPATION IN THE STUDY

I’M WILLING TO PARTICIPATE IN THE STUDY


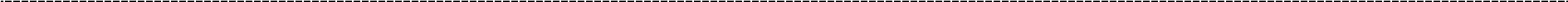


Place and date Signature participant


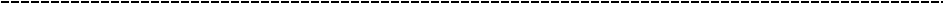


Participant name in capital letters

My contact information is:

Name____________________________________________________________________________________________

Date of birth____________________________________________________________________________________

Mobile/SMS ___________________________________________________________________________________

E-mail __________________________________________________________________________________________

Street__________________________________________________________________________________________

Postal code/city ______________________________________________________________________________

I confirm having received information about the study.


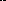

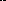

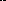

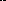

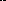

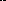

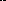

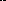

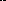

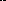

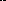

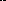

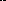

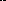

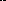

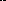

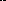

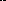

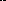

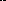

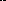

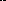

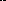

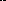

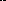

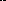

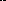

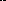

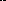

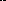

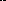

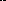

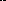

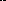

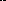

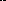

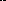

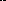

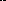

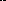

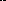

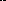

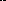

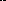

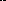

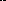

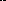

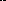

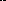

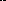

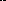

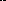

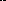

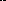

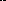

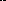

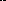

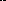

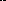

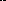

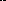

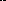

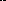

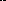

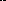

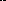

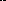

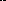

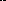

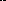

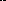

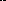

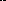

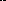

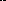

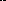

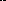

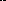

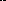

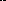

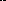

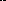

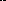

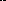

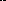

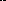

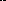

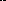

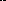

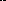

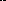

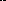

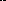

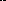

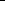

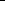


Place and date Signature

Role in project
