# Supplementary material for: Why do patients want medication free treatment for psychosis? An explorative study on reasons for applying to medication free programs
Source: BMC Psychiatry. 2024 Feb 16;24:127. doi: 10.1186/s12888-024-05513-9 (PMC10870549; doi:10.1186/s12888-024-05513-9)
Supplement: Supplementary file 5 — Additional file 5: About the reference group. [file 12888_2024_5513_MOESM5_ESM.docx]

**About the reference group**

Thinking that exploring the phenomenon would benefit from as much as possible, keeping and broaden perspectives on what to look for and how to understand what we find, we invited several clinicians, researchers and people with experience of having been patients.

They were invited and informed throughout the development of the project. At the time of writing there has been two formal meetings: In June 2022 there were a digital meeting followed by a workshop 18. November supplied with feedback from participants prohibited from participating.
