# Supplementary material for: Why do patients want medication free treatment for psychosis? An explorative study on reasons for applying to medication free programs
Source: BMC Psychiatry. 2024 Feb 16;24:127. doi: 10.1186/s12888-024-05513-9 (PMC10870549; doi:10.1186/s12888-024-05513-9)
Supplement: Supplementary file 6 — Additional file 6: Additional information about the population from which our sample is derived, that is; patients at the ward from January 2017 – October 2021. Information received from the Medication free ward quality register approved by Data Protection Official at University Hospital of North Norway (UNN) Number 02075. [file 12888_2024_5513_MOESM6_ESM.docx]

Additional information about *the population from which our sample is derived*, that is; patients at the ward from January 2017 – October 2021. Information received from the Medication free ward quality register approved by Data Protection Official at University Hospital of North Norway (UNN) Number 02075

They were mainly from the three counties (51,2 % from Troms County, 26,2 % from Nordland County, 20,2 % from Finnmark County and 2,4% other counties). Approximately 60 had been referred to treatment but excluded because of different reasons like withdrawal of application, in need of something else, not wanting this treatment, lack of supporting treatment-network.

At the time of referral (19) 22,6% had diagnoses of bipolar disorder, (55)65.6% of psychotic disorder and (10)11.9 % of other disorders treated with nevroleptic drugs. Before referral mostly all have been in contact with mental health services, many continuously as outpatient, ambulatory and day care services.

By October 2021, the number of admissions to the ward varied between 1-26 with average of 5 and 34,5% only had one admission. Length of admission varied from 1 day till more than a year. Most (90,5 %) admissions to the ward were elective. A few (9.5 %) had been offered acute admissions due to increased symptom pressure.

Many having experiences from both voluntarily treatment and coercion. At time of referral 50 (59,5%) were using neuroleptics on a regular basis. Many of they who did not using nevroleptics regularly, 34 (40,5 %), had ended medication on their own before attending. Less than 5 persons had never been using nevroleptics. Of those using nevroleptics at referral, 10 persons were by October 2021, tapered off medication, 14 were tapering off. (of the 10 finishing tapering off psychotropics four persons had finished treatment and six were still attending).

53 of 84 had at that time ended treatment. Nineteen of them were using nevroleptics upon discharge and most of them reported having reduces dosage during the time of treatment. A small percentage (2,4%) had unknown medical status and 4,8% had no plan for tapering off by discharge. Of the 53 persons, average admissions were 4,25 and this is quite similar to average for the whole group.

The population receiving invitation to participate in the study was N=79 fulfilling criteria having fulfilled one treatment admission, contact information available and being alive.
